# Pre-Sleep Alpha-Lactalbumin Supplementation Does Not Improve the Habitual Sleep and Performance of Sportspeople with Sleep Difficulties

**DOI:** 10.3390/nu17071196

**Published:** 2025-03-29

**Authors:** Jackson Barnard, Spencer Roberts, Michele Lastella, Brad Aisbett, Dominique Condo

**Affiliations:** 1 Centre for Sport Research, Institute of Physical Activity and Nutrition, Deakin University, Geelong 3220, Australia; s.roberts@deakin.edu.au (S.R.); dominique.condo@deakin.edu.au (D.C.); 2 Appleton Institute, Central Queensland University, Wayville 5034, Australia; m.lastella@cqu.edu.au; 3 School of Health, Medical and Applied Sciences, Central Queensland University, Rockhampton 4701, Australia; 4 Institute for Physical Activity and Nutrition, Deakin University, Geelong 3220, Australia; brad.aisbett@deakin.edu.au

**Keywords:** diet, protein, sleep, sport, trained

## Abstract

**Background**: Many athletes experience sleep difficulties, and prior research within this cohort suggests that acute supplementation of alpha-lactalbumin (ALAC), a whey protein rich in the amino acid tryptophan, may improve sleep and performance. Therefore, this study investigated whether sub-chronic ALAC supplementation in the evening would improve sleep and physical performance within a poor-sleeping athletic population. **Methods**: In total, 24 athletically trained participants with sleep difficulties (Athlete Sleep Screening Questionnaire: 8.6 ± 2.2; Pittsburgh Sleep Quality Index: 10.0 ± 3.0) completed this double-blinded, randomised controlled, crossover trial. The participants were supplemented with 40 g of ALAC or control 2 h pre-sleep for seven consecutive nights within habitual settings, with sleep measured via actigraphy. Performance was assessed following the 1-week supplementation period, with the 30 s countermovement jump test, Yo-Yo Intermittent Recovery Test Level 1, and reaction time testing performed in a standardised sequence under controlled conditions. **Results**: During the ALAC condition, the objective number of awakenings increased (CON: 10.25 ± 5.28, ALAC: 11.01 ± 5.79; *p* = 0.031), the average jump height reduced (CON: 28.58 ± 5.53 cm, ALAC: 27.68 ± 5.14 cm; *p* = 0.037), the subjective physical and mental performance capabilities declined in the evening (*p* < 0.001), and evening negative emotional states (*p* = 0.001) were reduced. **Conclusions**: Seven days of ALAC supplementation may not improve the sleep and physical performance of an athletically trained population with mild–moderate sleep difficulties. Future research should recruit populations with more severe sleep difficulties and measure sleep architecture over an extended period to fully ascertain the effects, and potential benefits, of ALAC supplementation for athletes.

## 1. Introduction

Cognitive and physical performance declines with poor sleep [1], a challenge faced by 50–78% of athletes experiencing sleep difficulties [2]. Though athletes may require more sleep than their non-athlete counterparts, factors such as pre-competition anxiety and training load contribute to the average athlete sleeping less than the general recommendation (i.e., 7–9 h) [2,3]. Poor sleep is associated with reduced sporting performance, particularly the degradation of cognitive and technical skills [1]. It is believed that, as sleep debt accumulates, extended physical performance is adversely affected [4]; therefore, poor-sleeping athletic populations are more vulnerable to significant performance decrements. Given that athlete-specific sleep barriers may prevent athletes engaging in, or limit the effectiveness of, good sleep hygiene, further investigation of practical strategies to improve sleep remains warranted.

An athlete’s diet influences their sleep, with the impact of protein sources high in the amino acid tryptophan (TRP) on sleep gaining interest [5]. Tryptophan appears to influence sleep by serving as a precursor to serotonin and melatonin synthesis—two primary hormones involved in sleep and wake regulation [6]. The downstream production of melatonin is limited by TRP’s availability to the brain, whereby availability is affected by the presence of other large neutral amino acids (LNAAs), given they share a common transporter to cross the blood–brain barrier [6]. The TRP:LNAA ratio is, therefore, an important determinant in TRP availability, whereby a 30–45% increase in the TRP:LNAA ratio is shown to increase brain serotonin production [7]. Evening supplementation of the highest-protein source of TRP, α-lactalbumin (ALAC; 48 mg TRP per g), increases the TRP:LNAA ratio up to 87% when consumed at 40 g, potentially leading to sleep and next-day performance improvements through the action of TRP [8,9,10].

The effect of ALAC supplementation on the sleep of athletic populations has been investigated in five studies [10,11]. Among these studies supplementing 40–60 g of ALAC in the evening, only the longest-duration trial (28 nights) demonstrated reductions in sleep latency [8]. Two single-day studies supplementing 40 g ALAC observed changes in sleep staging (i.e., NREM Stage 2 increase) [9,10], whilst two short-duration studies (<4 days) in well-trained cyclists observed no differences compared with control across all sleep metrics [12,13]. The previous TRP literature suggests that a loading effect may occur, with late-appearing reductions in sleep latency having occurred following three nights of 3 g TRP supplementation in sleep-onset insomniacs [14]. The possibility of a loading effect remains unexplored following ALAC supplementation, as only one study has supplemented ALAC for >3 consecutive nights [8]. Additionally, a ceiling effect may have occurred amongst acute ALAC studies recruiting ‘good sleepers’ [11], as the hypnotic effects of TRP-based supplementation are believed to be more pronounced in poor-sleeping populations [11,15].

Growing evidence suggests that evening supplementation of 40 g ALAC for 1–3 nights improves next-day cognition and physical performance [9,10]. Interestingly, these performance improvements occurred despite no changes in the sleep quality metrics, with increases only in NREM Stage 2 sleep occurring in both studies [9,10]. Given that TRP-based supplementation is believed to indirectly influence performance through changes in sleep quality and/or duration [16], the extent of these improvements should be further investigated. In addition, the real-world translation of ALAC on sleep and performance remains limited, as most studies have been conducted within laboratory settings [11]. Further, as longer ALAC supplementation periods are hypothesised to promote sleep quality more than acute supplementation, physical (e.g., ability to repeat multiple efforts) and cognitive performance tasks (e.g., reaction time) sensitive to changes in sleep may be further improved. Therefore, this study was the first to determine the efficacy of 40 g ALAC supplementation across seven days on the sleep, mood, and physical/cognitive performance of a sporting population with sleep difficulties within their habitual environment.

## 2. Materials and Methods

### 2.1. Participants

Twenty-four trained participants (females, *n* = 7) with sleep difficulties participated in this study (Table 1). The sample size was calculated using PASS software (version 16.0; NCSS, Kaysville, UT, USA), with sleep onset latency as the primary outcome [9,17]. A paired two-sided *t*-test was used to compare conditions in a 2 × 2 crossover design. A total sample of 24 participants provides 82% power to detect a mean difference of 13.0 min, assuming a standard deviation of 18.6 for the paired differences and a significance level of 0.05. All the participants were to be exercising ≥3 days per week for ≥5 h per week and training within an identified sport to comply with trained guidelines [18,19]. To meet sleep difficulty criteria, the participants required a sleep difficulty score ≥5 on the Athlete Sleep Screening Questionnaire (ASSQ), a global score >5 on the Pittsburgh Sleep Quality Index (PSQI), and a self-reported habitual sleep latency ≥15 min. The exclusion criteria were as follows: use of sleep medication, smoking, excessive alcohol consumption, dairy allergy, antidepressant medication use, a diagnosed sleep disorder, current or recent night shift work, and transmeridian travel within the past month. This study was approved by the Deakin University Human Research Ethics Committee (2021-416) and conducted in accordance with the Declaration of Helsinki.

### 2.2. Study Design

**Figure 1:**
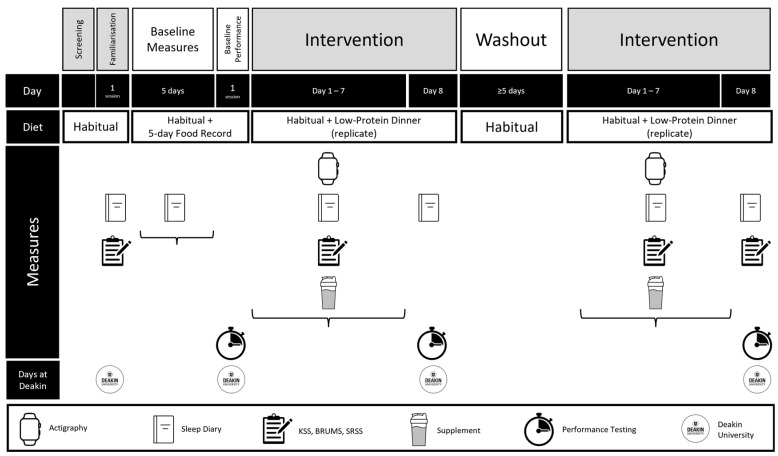
Graphic overview and timeline of this study. BRUMS = Brunel Mood Scale, KSS = Karolinska Sleepiness Scale, SRSS = Short Recovery Stress Scale in Sport.

This double-blinded, randomised controlled, counterbalanced, crossover experiment required participants to attend testing facilities at Deakin University on four occasions (Figure 1). Firstly, the participants completed a familiarisation session, during which they partially performed three physical performance tests sensitive to sleep changes (i.e., 30 s continuous jump test (CJ_30_), Yo-Yo Intermittent Recovery Test Level 1 (YYIR1), reaction time) [1]. Following this session, the participants recorded five consecutive days of sleep and food diaries, allowing the researchers to individually standardise sleep/wake times based on habitual averages during the experimental trials and observe typical eating behaviours. On a separate occasion post-familiarisation, the participants again reported to the testing facilities to fully perform the three physical performance tests to obtain baseline values.

### 2.3. Experimental Trials

The participants were randomised to either the ALAC or the control treatment for one week using block randomisation by an external researcher, wherein they consumed the supplement two hours prior to their individually prescribed bedtimes each night within their habitual setting [10]. Ninety minutes post-supplementation in the evening, the participants completed several questionnaires (i.e., Karolinska Sleepiness Scale (KSS), Brunel Mood Scale (BRUMS), Short Recovery Stress Scale in Sport (SRSS), training diary), as this timepoint is associated with peak sleepiness following TRP-based supplementation [20]. Similarly, questionnaires were completed in the morning (i.e., KSS, BRUMS, SRSS, sleep diary), 30 min after waking at the individually prescribed wake times. Following one week of supplementation, the participants attended testing facilities to perform the three physical performance tests, obtaining the post-treatment values.

After the completion of the first post-treatment physical performance tests, a minimum one-week washout phase followed during which no testing or supplementation occurred (22 ± 17 days), mitigating the risk of a carry-over effect following extended TRP-based supplementation [21]. Following the washout, the participants received the opposing supplement, with methods replicated for the second one-week treatment. Physical performance testing was again completed following the treatment period.

### 2.4. Dietary Supplement

The sequence of supplementation was allocated via simple randomisation in a counterbalanced manner by an external researcher. The experimental supplement consisted of 40 g ALAC (BiPRO Alpha 9000; Agropur Inc. Appleton, WI, USA), and the control contained 40 g of collagen (Collagen Hydrolysed Protein; True Protein, Brookvale, Australia), with the amino acid content of each supplement provided below (Table 2). Moreover, 15 g of sugar-free chocolate powder (Avalanche, Auckland, New Zealand) and 4 g of stevia (New Zealand Sugar Company, Auckland, New Zealand) were added to match the taste and texture [10]. Collagen was selected as the control supplement as it satisfied four primary criteria: minimal impact on the primary outcome of objective sleep latency [22], isocaloric, protein based, and low in TRP and other LNAAs.

The participants received a seven-day supply of supplements prior to each trial period within individual bags and were instructed to add ~400 mL of water to the supplement and consume these two hours prior to bedtime. To ensure adherence, an SMS reminder was sent to the participants at their individually prescribed consumption times via an automated service (ClickSend, Melbourne, Australia). All the participants verbally confirmed consumption of all supplements at post-treatment performance sessions.

### 2.5. Dietary Standardisation

The participants ate habitually throughout the trial; however, standardised pre-packaged dinner meals were provided each night, with an instruction to replicate their habitual diet across the trials. Diets were tracked for the entirety of the experimental trials via the smartphone application Easy Diet Diary (Xyris Software, v7.1; Brisbane, Australia), allowing the researchers to observe entries in real time. The diaries were assessed for inconsistencies and followed up by a sports dietitian prior to attending the post-treatment testing sessions. To reduce the confounding influence of protein on TRP availability, the provided dinner meals were primarily plant based (i.e., low in LNAAs) with lower protein (≤0.3 g·kg^−1^ bodyweight) and were to be consumed at least two hours prior to the supplement (≥4 h pre-sleep) [23]. Alongside the provided dinner meals (PRO: 14.9 ± 3.3 g [0.19 g/kg], CHO: 56.2 ± 13.8 g, FAT: 11.6 ± 9.9 g), the participants were permitted to consume only approved low-protein foods (e.g., rice, vegetables, olive oil) provided they were reported and replicated in the corresponding crossover period. Additionally, caffeine consumption was not permitted after 12:00 pm, with the timing and amount of caffeine replicated for each trial.

### 2.6. Sleep Assessment

The participants wore an actigraphy device (Actical Z; Phillips Respironics, Murrysville, PA, USA) on the wrist of their non-dominant hand each night of the experimental trials (i.e., seven nights). The actigraphy devices recorded activity in one-minute epochs, with analyses completed within device-specific software (Actiware; Phillips Respironics, Actiware v3.1) using a medium sensitivity threshold (>40 activity counts) [24]. In athletic populations, actigraphy displays good agreement (81–90%) and sensitivity (81–92%) with polysomnography for detecting sleep/wake measures [25]. The sleep variables extracted included the following:

- Bedtime (HH:MM): The self-reported clock time at which a participant attempts sleep.
- Waketime (HH:MM): The self-reported clock time at which a participant awakes for the final time and stops attempting sleep.
- Total sleep time (TST; min): The amount of sleep obtained between initial sleep onset and final awakening.
- Sleep efficiency (SE; %): The percentage of time in bed between bedtime and final wake time that was spent asleep.
- Sleep onset latency (SOL; min): The period between bedtime and initial sleep onset.
- Wake after sleep onset (WASO; min): The time spent awake after initial sleep onset and before final awakening.
- Awakenings (n): The number of wake periods detected after initial sleep onset.

### 2.7. Subjective Measures

Questionnaires were completed electronically at individually set times via electronic distribution software (REDCAP, v14.6; Research Electronic Data Capture, Nashville, TN, USA), with automated reminders sent at prescribed times to aid compliance.

### 2.8. Karolinska Sleepiness Scale

Sleepiness was assessed using the KSS, a Likert-type scale in which the number circled corresponds to the level of sleepiness within the preceding five minutes, ranging from 1 (extremely alert) to 9 (very sleepy, great effort keeping awake, fighting sleep) [26]. The KSS displays strong correlation with behavioural and EEG indicators of sleepiness [26].

### 2.9. Brunel Mood Scale

Mood was assessed using the BRUMS—a shortened version of the Profile of Mood States [27]. Twenty-four mood-based adjectives are rated on a five-point Likert scale in real time, ranging from not at all to extremely. Mood is then divided into six mood states (i.e., anger, confusion, depression, fatigue, tension, vigour), with scoring as per the original criteria [27]. This shortened mood scale displays moderate–high internal consistency, with Cronbach’s alpha ranging between 0.75 to 0.86 in young athletes [27].

### 2.10. Short Recovery Stress Scale for Sport

Subjective stress and recovery were measured using the SRSS, which contains eight scales individually rated from 0 (does not apply at all) to 6 (fully applies) in real time [28]. Four scales pertain to recovery (i.e., Physical Performance Capability, Mental Performance Capability, Emotional Balance, Overall Recovery), whilst the remaining four scales represent the current stress state (i.e., Muscular Stress, Lack of Activation, Negative Emotional State, Overall Stress). The SRSS has been validated within Australian sporting populations [28].

### 2.11. Consensus Sleep Diary

The Consensus Sleep Diary is a standardised sleep tool used to record subjective sleep metrics, including sleep/wake times, sleep latency, awakenings, and sleep quality using a five-point Likert scale [29]. The self-reported sleep/wake times were cross-referenced to confirm the sleep/wake states detected via actigraphy.

### 2.12. Training Diary

A training diary was completed each evening, detailing exercise type, start/finish time, and rating of perceived exertion (i.e., 0–10). Though the training sessions were habitual, the participants were to replicate their training sessions in each intervention period, which was confirmed by the researcher.

### 2.13. Physical Performance

Performance testing occurred following one week of each treatment within the Deakin University Sports Science Building. The participants performed the baseline and two post-treatment performance sessions at a the same approximate time of day, being either morning (9 am–12 pm, *n* = 10) or evening (5 pm–7 pm, *n* = 14) to simulate local sporting competition schedules and replication controlling for circadian effects on performance [30]. Further, the three performance tests were completed in a standardised sequence (i.e., warmup, CJ_30_, YYIR1, reaction time), with the time taken between each test self-determined (CJ_30_–YYIR1, 4.4 ± 1.0 min; YYIR1–reaction time, 9.0 ± 1.6 min) and replicated for each session.

### 2.14. The 30 s Continuous Jump Test (CJ_30_)

The participants were instructed to squat down until their knees were bent at an ~90-degree angle, have their hands on the hips, and perform maximal vertical jumps for 30 s [31]. The jumps were completed on portable force plates (type 9286; Kistler Instrumente AG, Winterthur, Switzerland), with the raw data imported to VALD software for analysis (ForceDecks v3.0; VALD, Brisbane, Australia). The average jump height, maximal jump height, number of jumps, and fatigue index were calculated. The fatigue index was calculated by considering the mean height of the first four jumps (H_First_4J_) and the end four jumps (H_End_4J_) in the following equation [31]:

$$\;\mathrm{Fatigue}\;\mathrm{Index}\;\left(\%\right)=\frac{H_{\mathrm{FIRST}\_4J}-H_{\mathrm{End}\_4J}}{H_{\mathrm{FIRST}\_4J}}\times 100$$

The CJ_30_ displays excellent test–retest reliability (ICC = 0.87) and adequately assesses anaerobic performance, especially within acyclic sports (e.g., basketball, volleyball) [31].

### 2.15. Yo-Yo Intermittent Recovery Test Level 1 (YYIR1)

The YYIR1 involved repeated 2 × 20 m runs between a starting, turning, and finishing line at progressively increased speeds as per audible beeps. The participants were required to walk/jog a 2 × 5 m recovery zone within a 10 s active rest period between each running bout. Testing was stopped once the participants failed to reach the finishing line twice consecutively, with the last completed level recorded. The YYIR1 strongly correlates with high-intensity running demands in team sports, including soccer (r = 0.71) [32].

### 2.16. Reaction Time

Using the Dynavision interactive light board (Dynavision Global Holdings LLC, Cincinnati, OH, USA), the participants performed activities across two modes: proactive and reaction time. The proactive mode involved pressing illuminated buttons for 60 s, with a new button lighting up following each press. The reaction time mode required participants to hold down a ‘home’ button with one hand, then hit a target button with the same hand once this button became lit. This was repeated 10 times for each hand per test, with three different tests completed (i.e., 30 times for each hand). Three results were calculated within the reaction time mode: visual reaction time (time taken to identify the target and release the home button), motor reaction time (time taken from release to pressing the target button), and physical reaction time (total time taken) [33]. Dynavision is effective for measuring visual-motor and visual-cognitive function as it strongly correlates with simple response time (*r* = −0.71) and choice response time (*r* = 0.65) tasks [34]—cognitive tasks sensitive to sleep changes [1].

### 2.17. Statistical Analysis

The researchers remained blinded to the treatment throughout the analyses. The data were checked for normal distribution and analysed using restricted maximum likelihood mixed models within StataBE 18 (StataCorp LLC, College Station, TX, USA). Dietary intervention (i.e., ALAC, control) was fitted as a fixed effect to determine whether there was a difference in the effect of dietary intervention over a period on dependant sleep variables, mood, sleepiness, recovery, and performance measures across periods. Participant identification numbers were included as a random factor to account for repeated measures within each model, with the repeated degrees of freedom method utilised to improve accuracy within smaller samples. All the variables are reported as mean ± SD, with significance set at *p* < 0.05.

## 3. Results

Twenty-five participants started this study, with one participant withdrawing during the first trial for personal reasons (96% retention rate, *n* = 24). The participants competed in a variety of sports (e.g., Australian rules football, jiujitsu, soccer) mostly at a local level. The habitual macronutrient (CHO: 2.8 ± 0.9; FAT: 1.0 ± 0.3; PRO: 1.2 ± 0.4 g/kg) and energy consumption (107.4 ± 28.3 kJ/kg) was similar between treatments.

### 3.1. Sleep

#### 3.1.1. Objective Sleep

Eighty-five percent of the sleep recordings were included in the analysis, with nights on which sleep/wake times could not be clearly identified removed (i.e., incomplete sleep diaries and/or participants not wearing the watch). Compared with control, the number of awakenings were increased in the ALAC condition (*p* = 0.031). No treatment effects were observed for any other objective sleep metrics (Table 3).

#### 3.1.2. Subjective Sleep

A positive treatment effect was observed in the ALAC condition for subjective number of awakenings (*p* = 0.041). No differences were observed for any other subjective sleep metric (Table 3).

### 3.2. Questionnaires

#### 3.2.1. Sleepiness and Mood

There were no significant differences across treatments for sleepiness or any mood states in the morning or evening period (*p* > 0.05).

#### 3.2.2. Recovery and Stress

There were no differences between treatments for any recovery or stress state measured in the morning (*p* > 0.05). In the evening, however, during the ALAC condition, reductions in negative emotional state (*p* = 0.001), physical performance (*p* = 0.004), mental performance (*p* < 0.001), and emotional balance (*p* = 0.026) were observed compared with control (Table 4).

### 3.3. Performance Measures

#### 3.3.1. The 30 s Continuous Jump Test (CJ_30_)

The average jump height was reduced in the ALAC condition compared with control (*p* = 0.037); however, no significant difference was observed in any other jumping outcomes (Table 5). Compared with the baseline, the control trial had an increase in total jumps (*p* = 0.003) and total jump load (*p* = 0.009), with a reduced maximum jump height (*p* = 0.041). Post-ALAC, the total jump load increased (*p* = 0.018) and the average jump height decreased (*p* = 0.018) compared with baseline measures.

#### 3.3.2. Yo-Yo Intermittent Recovery Test Level 1

There were no differences between treatments for YYIR1 score (CON: 22.05 ± 11.40; ALAC: 21.87 ± 11.97, *p* = 0.404) or distance (*p* = 0.375). The baseline measures were not different from the post-treatment values.

#### 3.3.3. Dynavision Lightboard

Across both the proactive and reaction time modes, no significance was observed across treatments (*p* > 0.08) or post-treatment values compared with the baseline (*p* > 0.05).

## 4. Discussion

This study examined the effects of a pre-sleep supplement (40 g ALAC) on the sleep, mood, recovery, and physical performance of sportspeople with sleep difficulties in habitual settings. The main findings were that sleep quality, sleep duration, and physical performance remained mostly unchanged compared with control; however, there were increased markers of evening fatigue and a reduction in the average jump height following the ALAC supplementation. These findings contradicted the hypothesis that ALAC would promote sleep initiation, maintenance, and physical performance amongst a poor-sleeping sporting population.

Contrary to the previous data [8], 40 g of ALAC supplemented in the evening did not improve sleep quality and, in fact, led to more awakenings than control. Whilst the number of awakenings were slightly increased in the ALAC condition (+0.76; *p* = 0.031), importantly, this did not lead to an increase in WASO or a reduction in sleep efficiency and duration. This minor difference in awakenings may be explained by the reduced capacity of actigraphy to detect wake periods in poor-sleeping individuals, with sleep often underestimated in populations exhibiting more movement [2,35]. Although extended sleep monitoring using actigraphy (≥5 days) is observed to reduce error and increase reliability [35], this may still have resulted in minor changes. With the recent ALAC literature suggesting that sleep architecture may be altered following acute supplementation in controlled environments [9,10], future field studies should look to utilise more comprehensive sleep tools (e.g., portable electroencephalography) to observe this trend over a longer duration and to confirm the potential impact on awakenings.

The effects of ALAC may be more pronounced amongst populations with severe sleep difficulties. At screening, the average participant had moderate sleep difficulties (ASSQ 8.6), with a self-reported sleep latency of 30.3 min. Whilst sleep latency values >30 min are not considered good quality sleep by the National Sleep Foundation, the actigraphy-derived latency values measured throughout this study were (<17 min) [3]. This indicates that future studies investigating poor sleepers should confirm sleep difficulties through objective means, as the subjective latencies at baseline were likely overestimated. Additionally, as actigraphy may underestimate sleep latency in athletes by ~9.5 min [24], recruiting populations with more severe sleep onset difficulties—such as those defined by the National Sleep Foundation (i.e., ≥45 min)—may be necessary to detect meaningful differences in actigraphy-derived sleep latency values following ALAC supplementation. This concept is supported by the only athlete-based ALAC study to report reductions in sleep latency [8], with their participants displaying the longest baseline sleep latency values compared with other studies [10,11].

Alpha-lactalbumin supplementation demonstrated greater subjective indicators of evening fatigue than control. Though there were no changes to sleepiness reported within the KSS, compared with control, reduced evening states of physical performance (*p* = 0.004), mental performance (*p* < 0.001), and negative emotions (*p* = 0.001) were recorded in the SRSS during the ALAC trial. An increase in the TRP:LNAA ratio following ALAC supplementation has been previously accompanied by reduced depressive feelings in stressed subjects [15], aligning with the reductions in negative emotional state observed within this study. Interestingly, however, emotional balance was also reduced (−0.22; *p* = 0.026), indicating that whilst the participants felt fewer negative emotions, their ability to regulate emotions may have been hindered. A reduced ability to regulate emotions is associated with increased fatigue, whereby emotional intensity and balance is impaired during periods of reduced mental performance capacity [36]—though this was not apparent within the BRUMS responses. Nonetheless, the findings that negative emotional states were reduced may be particularly appealing to athletes who struggle with pre-competition anxiety. Despite actigraphy-derived sleep latency and efficiency being unchanged following ALAC supplementation in this study, future investigations should be completed during times of increased competition-related stressors (and, thus, potentially increased sleep disturbance) to further understand this effect.

The physical performance tests were not improved following one week of ALAC supplementation. Whilst the average jump height in the CJ_30_ was higher in the control group (−0.90 cm; *p* = 0.037), there were no differences in the total jump load (*p* = 0.733) between trials. Although reductions in the physical performance capability were only reported in the ALAC trial pre-sleep, subjective fatigue may have carried over into the next-day performance testing. This finding appears more psychological than mechanical, given that short-term ALAC supplementation is superior to the control (i.e., collagen) for enhancing myofibrillar and sarcoplasmic protein synthesis [13]. Additionally, whilst the reduction in the average jump height may relate to the increased number of awakenings observed within the ALAC trial, this study was powered for sleep latency, thereby, detecting meaningful changes in exploratory variables due to awakenings may require larger sample sizing. Further, no differences were observed in the post-treatment YYIR1 testing, even though this has been improved following ALAC previously [9]. Tryptophan-based supplementation is not ergogenic in itself [16], whereby performance improvements are believed to be mediated through sleep changes. This may explain the minor effects on performance in this study, as the selected performance tests involved components influenced through sleep quality and duration [1]. Future studies should assess recovery states immediately preceding physical performance testing and measure sleep architecture over an extended duration to elucidate the true efficacy and mechanistic impact of ALAC on performance.

The applied nature of this study is a strength, with the participants residing within their habitual settings and performing sports-related performance tasks. Low-protein dinners were provided to participants each night, limiting the effect that LNAA intake has on ALAC efficacy in the evening [23]. Though a habitual diet was replicated across both crossover periods (PRO: 1.2 ± 0.4 g/kg), the extent to which the daily protein intakes influence sleep and the evening TRP:LNAA ratio remains unclear, with further research also required to confirm the effect of the total dietary TRP intake throughout the day. To increase compliance, reminders were sent at individually prescribed times for supplement consumption, questionnaire completion, and to wear the actigraphy device. Although actigraphy is a valid tool to measure the sleep of athletic populations [25], an inability to detect sleep staging and the accuracy of sleep latency and awakening metrics warrants further research utilising more comprehensive measures [24]. Furthermore, the relatively short supplementation period (i.e., seven nights) limits the ability to assess the long-term effects of ALAC on sleep, highlighting the need for longer-duration studies to better understand its efficacy in trained populations with sleep difficulties. Lastly, due to the complexity of scheduling, the sleep and performance measures could not be collected solely within a specific menstrual phase. Although underpowered for sex comparisons, an exploratory analysis of the data for each sex was not different for sleep and subjective outcomes (*p* > 0.05), with physical performance outcomes trending similarly for females. While this approach cannot completely exclude menstrual cycle effects on outcomes, the data suggest that the menstrual cycle has a minimal impact on objective sleep (i.e., latency, efficiency) [37] and physical performance [38].

## 5. Conclusions

This is the first study to investigate the effect of 40 g α-lactalbumin supplementation in the evening on the sleep, mood, and next-day physical performance in sportspeople with sleep difficulties. In the ALAC condition, minor negative effects were noted in sleep (i.e., awakenings) and performance (i.e., average jump height), with increased indicators of fatigue and reduced negative emotional state observed in the evening. Alpha-lactalbumin may not be more effective than control on the sleep and performance of an athletic population with mild–moderate sleep difficulties; however, future field-based studies supplementing ALAC should focus on athletic populations with severe sleep difficulties (i.e., sleep latency >45 min).

### Practical Applications

- Overall, 40 g α-lactalbumin two hours before bedtime does not impair sleep efficiency or duration over one week.
- Negative emotional states may be reduced following ALAC consumption in the evening.
- Pre-sleep subjective mental and physical performance capability may be reduced post-ALAC consumption.
- Future sleep studies within trained populations with sleep difficulties should opt for more comprehensive sleep tools (e.g., portable electroencephalography) and objectively confirm baseline sleep difficulties.

## Figures and Tables

**Table 1:** Participant demographic and characteristic details.

Variable	Mean ± SD
Age (years)	25.9 ± 5.3
Body mass (kg)	80.0 ± 10.6
Height (m)	1.8 ± 0.1
ASSQ (sleep difficulty score)	8.6 ± 2.2
PSQI (global score)	10.0 ± 3.0
Subjective sleep latency (min)	30.3 ± 16.4

**Table 2:** Large neutral amino acid composition per 40 g of protein supplements.

Large Neutral Amino Acids (LNAAs)	α-Lactalbumin (40 g)	Control— Collagen (40 g)
Isoleucine (g)	2.4	0.5
Leucine (g)	4.3	1.0
Phenylalanine (g)	1.6	0.6
Tyrosine (g)	1.8	0.2
Valine (g)	1.7	0.9
Tryptophan (g)	1.9	0.0

**Table 3:** Objective and subjective sleep metrics.

Sleep Metric	CON	ALAC	*p* Value	Treatment Coefficient	95% CI	
Actigraphy-derived metrics						
TST (h)	7.26 ± 1.06	7.12 ± 1.04	0.084	−0.139	−0.297	0.019
SOL (min)	16.53 ± 15.51	16.70 ± 13.34	0.932	0.128	−2.834	3.089
WASO (min)	34.69 ± 21.14	37.58 ± 23.20	0.075	3.191	−0.328	6.711
SE (%)	88.77 ± 5.20	87.96 ± 5.32	0.077	−0.838	−1.767	0.091
Awakenings (#)	10.25 ± 5.28	11.01 ± 5.79	0.031 *	0.846	0.078	1.613
Subjective sleep metrics						
Bedtime (HH:MM)	22:45 ± 01:09	22:54 ± 01:25	0.237	0.005 (00:07)	−0.003 (−00:04)	0.013 (00:18)
Waketime (HH:MM)	07:02 ± 01:32	07:08 ± 01:39	0.541	0.002 (00:03)	−0.005 (−00:08)	0.010 (00:15)
sSOL (min)	18.98 ± 18.43	21.31 ± 22.81	0.153	2.519	−0.944	5.983
sWASO (min)	9.56 ± 20.72	12.91 ± 26.51	0.165	3.530	−1.459	8.519
Awakenings (#)	1.52 ± 1.38	1.81 ± 1.69	0.041 *	0.266	0.011	0.521
Sleep Quality (1–5)	3.34 ± 0.83	3.36 ± 0.85	0.785	0.023	−0.143	0.189

Note. The data used for analysis are the means of Nights 1–7. ALAC (α-lactalbumin), CI (confidence interval), CON (control), SE (sleep efficiency), sSOL (subjective sleep onset latency), SOL (sleep onset latency), sWASO (subjective wake after sleep onset), TST (total sleep time), WASO (wake after sleep onset). * Significantly different compared with control (*p* < 0.05).

**Table 4:** Subjective stress and recovery states in the evening.

Recovery/Stress State	CON	A-LAC	*p* Value	Treatment Coefficient	95% CI	
Evening						
Muscular Stress	2.48 ± 1.40	2.35 ± 1.50	0.375	−0.119	−0.381	0.144
Lack of Activation	2.21 ± 1.38	2.15 ± 1.33	0.661	−0.050	−0.276	0.176
Negative Emotional State	1.41 ± 1.33	1.04 ± 1.08	0.001 *	−0.331	−0.527	−0.136
Overall Stress	2.20 ± 1.37	2.26 ± 1.36	0.572	0.068	−0.170	0.306
Physical Performance	3.16 ± 1.27	2.84 ± 1.26	0.004 *	−0.327	−0.549	−0.106
Mental Performance	3.18 ± 1.47	2.77 ± 1.16	<0.001 *	−0.422	−0.637	−0.207
Emotional Balance	3.68 ± 1.40	3.46 ± 1.25	0.026 *	−0.252	−0.474	−0.031
Overall Recovery	3.16 ± 1.27	2.99 ± 1.27	0.118	−0.186	−0.419	0.047

Note. The data used for analysis are the means of Nights 1–7. ALAC (α-lactalbumin), CI (confidence interval), CON (control). * Significantly different compared with control (*p* < 0.05).

**Table 5:** The 30 s continuous jump test.

CJ_30_ Outcome	Baseline	CON	ALAC	*p* Value	Treatment Coefficient	95% CI	
Total jumps (n)	19.74 ± 4.73	21.39 ± 5.06 ^#^	21.83 ± 4.73	0.458	0.435	−0.759	1.629
Maximum jump height (cm)	33.36 ± 6.25	32.16 ± 5.73 ^#^	32.26 ± 5.59	0.836	0.010	−0.889	1.089
Average jump height (cm)	28.72 ± 5.55	28.58 ± 5.53	27.68 ± 5.14 ^#^	0.037 *	−0.907	−1.753	−0.061
Total jump load (cm)	550.96 ± 116.22	595.80 ± 128.91 ^#^	590.15 ± 122.31 ^#^	0.733	−5.659	−39.605	28.286
Fatigue Index (%)	16.62 ± 10.88	15.90 ± 8.30	20.36 ± 10.85	0.059	4.461	−0.179	9.101

Note. The data used for analysis are the means of Nights 1–7. ALAC (α-lactalbumin), CI (confidence interval), CJ_30_ (30 s continuous jump test), CON (control). * Significantly different compared with control (*p* < 0.05). ^#^ Significantly different compared with baseline (*p* < 0.05).

## Data Availability

The original contributions presented in the study are included in the article, further inquiries can be directed to the corresponding author/s.

## Abbreviations

The following abbreviations are used in this manuscript:

ALAC	Alpha-lactalbumin
ASSQ	Athlete Sleep Screening Questionnaire
BRUMS	Brunel Mood Scale
CJ_30_	30 s countermovement jump test
KSS	Karolinska Sleepiness Scale
LNAA	Large neutral amino acid
NREM	Non-rapid eye movement
PSQI	Pittsburgh Sleep Quality Index
SRSS	Short Recovery Stress Scale in Sport
TRP	Tryptophan
YYIR1	Yo-Yo Intermittent Recovery Test Level 1
